# Charge transport mechanism in dielectrics: drift and diffusion of trapped charge carriers

**DOI:** 10.1038/s41598-020-72615-1

**Published:** 2020-09-25

**Authors:** Andrey A. Pil’nik, Andrey A. Chernov, Damir R. Islamov

**Affiliations:** 1 Rzhanov Institute of Semiconductor Physics, Siberian Branch of the Russian Academy of Sciences, Novosibirsk, 630090 Russian Federation; 2 Novosibirsk State University, Novosibirsk, 630090 Russian Federation

**Keywords:** Electronic devices, Electronic and spintronic devices

## Abstract

In this study, we developed a continuum theory of the charge transport in dielectrics by trapped electrons and holes, which takes into account two separate contributions of the current of trapped charge carriers: the drift part and the diffusion one. It was shown that drift current is mostly dominant in the bulk, while the diffusion one reaches significant values near contacts. A comparison with other theoretical models and experiments shows a good agreement. The model can be extended to two- and three-dimensional systems. The developed model, formulated in partial differential equations, can be numerically implemented in the finite element method code.

## Introduction

The knowledge about charge transport processes and mechanisms in dielectrics is critical for modern microelectronics, because many dielectrics, like $\hbox {SiO}_2$ and high-$\kappa$ (for example $\hbox {HfO}_2$) are used as gate dielectrics^1,2^ and low-$\kappa$ ones—as insulating dielectrics separating the wire interconnects and transistors from each other in high-speed integrated circuits^3^, as well as blocking insulators in silicon-oxide-nitride-oxide-silicon-type (SONOS)^4,5^ and TaN-high-$\kappa$-nitride-oxide-silicon-type (TANOS)^6,7^ flash memory devices. High-$\kappa$ dielectrics are promising candidates to be used as functional materials in resistive random access memory devices (RRAM) and memristors, that would involve integrating the most favourable properties of both rapid dynamic random access memory (DRAM) and non-volatile solid drive memory (SSD)^8–12^. Therefore, MOSFETs and TANOS need high-$\kappa$ and low-$\kappa$ dielectrics featuring low leakage currents, but RRAM cells require a dielectric medium that exhibits reversible resistive switching. Controlling the process of dielectric film synthesis to manage leakage currents can help creating high-end components for different devices.

In general, the charge transport mechanisms in dielectric layers can be conditionally divided into two groups: contact-limited and bulk-limited via traps ones. The first group is the contact-limited models that describe electrons and holes emission from metal or semiconductor contacts to the conduction band or traps in dielectric layers. The field emission, also known as Fowler–Nordheim model, describes charge carriers tunnelling through a triangle energy barrier to the dielectric conduction band^13^. This is the quantum effect that does not depend on the temperature. As far as the Fermi energy level, as well as the electron energy distribution in metals and semiconductor contacts, depends on temperature, thermally assisted tunnelling might give a significant contribution to the current^14,15^. At the first stage, charge carriers are excited to a certain energy due to the phonon absorption, and then they tunnel through the triangular barrier. In case of high temperatures, the field-enhanced thermionic emission (Schottky effect) of hot carriers takes place^16^. A combination of Fowler–Nordheim tunnelling with the Schottky effect gives the so-called Schottky–Nordheim barrier, which is the barrier model used in deriving the standard Fowler–Nordheim-type equation^17^. In case of ultrathin ($<5\,\hbox {nm}$) dielectric films, charge carriers might exhibit their direct tunnelling from one electrode to another through a trapeze barrier^18^. If the structure is based on a thick ($>5\,\hbox {nm}$) dielectric layer with traps, then the electrons and holes might be injected within the trap-assisted tunnelling (TAT) mechanism^19^. At the first stage, charge carriers tunnel from the first electrode to the trap in the dielectric film bulk, and then they tunnel from the trap to the conduction (valence) band in the next electrode. Such a multi-stage process may be more profitable than direct tunnelling or the tunnelling according to the Fowler–Nordheim mechanism.

The first theoretical bulk-limited charge transport model was proposed by Frenkel^20,21^. This model is based on a single charged Coulomb centre (trap). In a strong electric field the Coulomb barrier is decreased, and a localized electron (hole) can leave the trap through three channels: by tunnelling through a reduced energy barrier, by the thermally assisted tunnelling through the barrier and by the thermal emission over a barrier. In case of closed traps, the Coulomb centres overlapping additionally decreases the barrier between traps (Hill-Adachi model)^22,23^, and the current-voltage characteristics correspond to Poole law^24,25^. Recently, it has been demonstrated, that phonons play the key role in the charge transport processes in dielectrics. The TAT model for ultrathin dielectrics has been extended with the involvement of multiphonon processes as the trap-assisted inelastic tunnelling^26,27^. The multiphonon single trap ionization model for thick dielectric films was proposed by Makram-Ebeid and Lannoo^28^. This model is based on a $\delta$-like neutral quantum well with a single energy level. The trapped electron with phonons interaction leads to an increase in the probability of tunnel trap ionization to the conduction band of the dielectric. In case of closed traps, the charge carriers couple with phonons exhibit the tunnelling between traps without ionization to the conduction (valence) band. This is the model of phonon-assisted tunnelling of electrons (holes) between neighbouring traps (PATENT)^29^.

Despite the large number of contributions in the literature, an unresolved issue is describing leakage currents by trapped charge carriers (electrons or holes) on deep centres in dielectrics in terms of two- and three-dimensional continuum models. Earlier, a scheme of linear differences was used to calculate the trapped charge carrier distribution in dielectric film-based devices^29–31^.

## Results

### Direct simulations

For simplicity, the tunnelling current through dielectrics can be reduced to the tunnelling-driven movement of charges along the multiple independent chains of traps. The probability of tunnelling between traps heavily depends on the spacing between them and electric field and may be different for each pair. Considering only the tunnelling between the nearest traps and assuming tunnelling to distant traps to be highly improbable, the evolution of a chain of traps can be described by the following equations:

1 $$\begin{aligned} {\text {P}}_i^+&= \delta t \left[ {\text {Tun}}_{i-1,i}^+ n_{i-1} + {\text {Tun}}_{i,i+1}^- n_{i+1}\right] \left( 1 - n_i\right) \\ {\text {P}}_i^-&= \delta t \left[ {\text {Tun}}_{i-1,i}^- (1 - n_{i-1}) + {\text {Tun}}_{i,i+1}^+ (1 - n_{i+1})\right] n_i, \end{aligned}$$

where ${\text {P}}_i^+$ is the probability of charge trapping on the $i{{\text{th}}}$ trap in an infinitesimal amount of time $\delta t$; ${\text {P}}_i^-$ is the probability of charge leaving the $i{{\text{th}}}$ trap in an infinitesimal amount of time $\delta t$; $n_i$ is the occupancy of the $i{{\text{th}}}$ trap ($n_i = 1$ if a trap is occupied with a charge carrier and $n_i = 0$ if a trap is empty); ${\text {Tun}}_{i, j}^\pm$ is the probability rate of tunnelling between the $i{{\text{th}}}$ trap and $j{{\text{th}}}$ trap (superscripts ‘$+$’ and ‘−’ represent forward and backward tunnelling, respectively). The evolution of *M*-trap chain endpoint traps is described by the following equations:

2 $$\begin{aligned} {\text {P}}_1^+&= \delta t \left[ {\text {Inj}}_1 + {\text {Tun}}_{1,2}^-n_2\right] \left( 1 - n_1\right) \\ {\text {P}}_1^-&= \delta t \left[ {\text {Ion}}_1 + {\text {Tun}}_{1,2}^+ \left( 1 - n_2\right) \right] n_1\\ {\text {P}}_{M}^+&= \delta t \left[ {\text {Inj}}_M + {\text {Tun}}_{M-1,M}^+n_{M-1}\right] \left( 1 - n_M\right) \\ {\text {P}}_{M}^-&= \delta t \left[ {\text {Ion}}_M + {\text {Tun}}_{M-1,M}^-\left( 1 - n_{M-1}\right) \right] n_M ,\\ \end{aligned}$$

where ${\text {Inj}}_i$ is the probability rate of charge injection from the electrode into the $i{{\text{th}}}$ trap, and ${\text {Ion}}_i$ is the probability rate of ionization and subsequent removing into an electrode of a charge carrier from the $i{{\text{th}}}$ trap.

Considering the occupancy of neighbouring traps to be independent random values, the evolution of this value is given by the following equation:

3 $$\begin{aligned} \frac{\text{d}{\bar{n}}_{i}}{\text{d}t} = \left[ {\text {Tun}}_{i-1,i}^+ {\bar{n}}_{i-1} + {\text {Tun}}_{i, i+1}^{-} {\bar{n}}_{i+1}\right] (1 - {\bar{n}}_{i}) - \left[ {\text {Tun}}_{i-1, i}^{-} (1 - {\bar{n}}_{i-1}) + {\text {Tun}}_{i,i+1}^+ (1 - {\bar{n}}_{i+1})\right] {\bar{n}}_{i}, \end{aligned}$$

where ${\bar{n}}_{i}$ is the average occupancy of the $i{{\text{th}}}$ trap. The evolution of average occupancy of the traps, placed at the chain endpoints is described by the following equations:

4 $$\begin{aligned} \frac{\text{d}{\bar{n}}_1}{\text{d}t}&= \left[ {\text {Inj}}_1 + {\text {Tun}}_{1,2}^-{\bar{n}}_2\right] (1 - {\bar{n}}_1) - \left[ {\text {Ion}}_1 + {\text {Tun}}_{1,2}^+ (1 - {\bar{n}}_2)\right] {\bar{n}}_1 \\ \frac{\text{d}{\bar{n}}_{M}}{\text{d}t}&= \left[ {\text {Inj}}_M + {\text {Tun}}_{M-1,M}^+{\bar{n}}_{M-1}\right] (1 - {\bar{n}}_{M}) - \left[ {\text {Ion}}_M + {\text {Tun}}_{M-1,M}^- (1 - {\bar{n}}_{M-1})\right] {\bar{n}}_{M}, \end{aligned}$$

**Figure 1:**
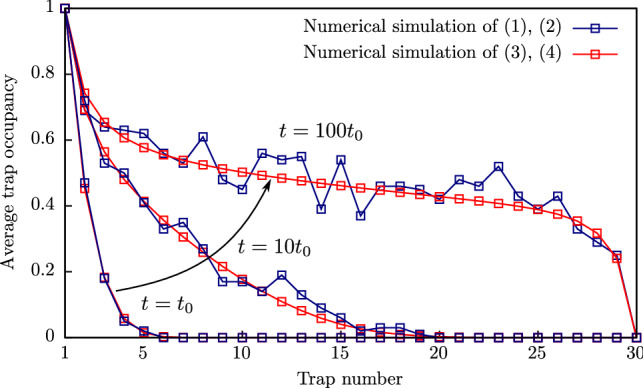
Average occupancy of traps in a 30-trap chain at different moments obtained by calculating Eqs. (1) and (2) for 500 chains and by calculating Eqs. (3) and (4).

**Figure 2:**
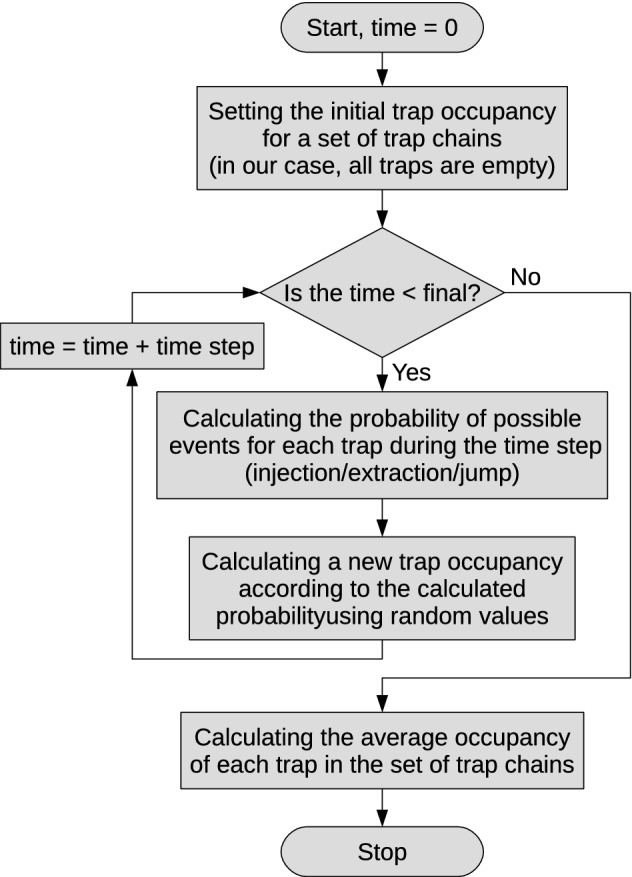
The flowchart of simulations based on Eqs. (1) and (2).

Figure 1 shows a numerical (averaged over 500 calculations) solution of Eqs. (1) and (2) by blue symbols, a numerical solution of Eqs. (3) and (4) by red symbols, obtained at different moments for the 30-trap chain in case of bulk-limited current. The flowchart for the (1) and (2)-based simulations is shown on Fig. 2. Equations (3) and (4) are solved within standard integration techniques. All simulation parameters are shown in Table 1. One can see that both approaches give the solutions that are in a good agreement with each other. The tunnelling rate is considered to be the same for any pair of neighbouring traps so that ${\text {Tun}}^+_{i, i+1} = P$. Hereinafter, $t_0 = 1/P$ is the latency time of tunnelling from an occupied trap to an empty neighboring one.

**Table 1:** Parameters used in the direct simulations.

${\text {Tun}}_{i,j}^-$	${\text {Tun}}_{i,i+1}^+$	$t_0$	*M*	${\bar{n}}_1$	${\bar{n}}_M$
0	*P* (any, e.g. 1)	1/*P*	30	1	0

In the case of bulk-limited current, Eq. (4) can be rewritten simply as: ${\bar{n}}_1 = 1$, ${\bar{n}}_{M} = 0$. This scheme is applicable as it is and it was used by different authors^29–31^, but it has a number of limitations. First of all, it scales badly with the increase in the number of traps (computing the scheme for a large number of traps can require great computational power). Also, the use of analytical considerations and conventional numerical analysis dictates the need for the formulation of the problem in the form of a system of partial differential equations.

### Continuum model

Probability rates ${\text {Inj}}_i$ and ${\text {Ion}}_i$ depend on the temperature and electric field at the $i{{\text{th}}}$ trap and can be considered a function of the $i{{\text{th}}}$ trap coordinate $x_i$. By the same logic, ${\text {Tun}}^\pm _{i, i+1}$ can be considered a function of coordinate $(x_{i+1} + x_i)/2$ halfway between traps. By considering ${\bar{n}}$ to be a continuous function of time and coordinate and using the second order Taylor expansion of ${\bar{n}}_{i+1}$, ${\bar{n}}_{i-1}$, ${\text {Tun}}^+_{i-1,i}$, ${\text {Tun}}^-_{i-1,i}$, ${\text {Tun}}^+_{i,i+1}$, ${\text {Tun}}^-_{i,i+1}$ around the $i{{\text{th}}}$ trap in Eq. (3), the following partial differential equation can be obtained after factoring resulting members:

5 $$\begin{aligned} \frac{\partial {\bar{n}}}{\partial t} = - a \frac{\partial }{\partial x} \left\{ \left[ {\text {Tun}}^+ - {\text {Tun}}^-\right] {\bar{n}}\left( 1 - {\bar{n}}\right) \right\} +\dfrac{a^2}{2} \frac{\partial }{\partial x} \left\{ \left[ {\text {Tun}}^+ + {\text {Tun}}^-\right] \frac{\partial {\bar{n}}}{\partial x}\right\} , \end{aligned}$$

where *x* is the coordinate; ${\text {Tun}}^\pm = {\text {Tun}}(\pm F)$, *F* is the electric field and *a* is the distance between traps. The boundary conditions are determined by the current continuity at the boundaries and can be described by the following equations:

6 $$\begin{aligned} \frac{\partial {\bar{n}}}{\partial x}\Big |_{x = +0}&= \dfrac{2}{a} \dfrac{{\text {Tun}}^+ - {\text {Tun}}^-}{{\text {Tun}}^+ + {\text {Tun}}^-}{\bar{n}}(1 - {\bar{n}}) -\dfrac{2}{a} \dfrac{{\text {Inj}}(1 - {\bar{n}}) - {\text {Ion}}{\bar{n}}}{{\text {Tun}}^+ + {\text {Tun}}^-}\Big |_{x = +0};\\ \frac{\partial {\bar{n}}}{\partial x}\Big |_{x = d-0}&= \dfrac{2}{a} \dfrac{{\text {Tun}}^+ - {\text {Tun}}^-}{{\text {Tun}}^+ + {\text {Tun}}^-}{\bar{n}}(1 - {\bar{n}}) +\dfrac{2}{a} \dfrac{{\text {Inj}}(1 - {\bar{n}}) - {\text {Ion}}{\bar{n}}}{{\text {Tun}}^+ + {\text {Tun}}^-}\Big |_{x = d-0}. \end{aligned}$$

The dependence of functions ${\text {Inj}}$ and ${\text {Ion}}$ on the values of temperature and electric field at a given coordinate. Current density values can be obtained using the charge density continuity equation and written as:

7 $$\begin{aligned} j = \underbrace{q N a \left[ {\text {Tun}}^+ - {\text {Tun}}^-\right] {\bar{n}}(1 - {\bar{n}})}_{j_{\text {drift}}} \,\,\underbrace{- q N \dfrac{a^2}{2} \left[ {\text {Tun}}^+ + {\text {Tun}}^-\right] \frac{\partial {\bar{n}}}{\partial x}}_{j_{\text {diff}}}, \end{aligned}$$

where *N* is the trap density, *q* is the elementary charge (absolute value). Due to the structure of Eq. (7), it is convenient to treat the first part of the right-hand side of the equation as a ‘drift’ current $j_{{\text {drift}}}$ and the second part—as a ‘diffusion’ current $j_{{\text {diff}}}$. It is important to note that, although the diffusion current is proportional to the higher order of small parameter *a*, it cannot be omitted. This fact can be easily seen from the following illustrative example. In the case of uniform tunnelling probability for every trap, Eq. (5), obviously, has no non-trivial stationary solution. Contrariwise, the solution of the full form of Eq. (5) can be found analytically. For example, in the case of bulk-limited current (${\bar{n}}(0) = 1$, ${\bar{n}}(d) = 0$, where *d* is the chain length), the solution is:

8 $$\begin{aligned} {\bar{n}}({\tilde{x}}) = 1/2 \left\{ 1 - \Omega \beta (\Omega ) \tan \left( \beta (\Omega )\left[ 2{\tilde{x}} - 1\right] \right) \right\} , \end{aligned}$$

where

$$\begin{aligned} \Omega = 2\dfrac{a}{d} \dfrac{{\text {Tun}}^+ + {\text {Tun}}^-}{{\text {Tun}}^+ - {\text {Tun}}^-} \end{aligned}$$

is the dimensionless number that characterizes the trapped charge spatial distribution in the stationary state; ${\tilde{x}} = x/d$ is the dimensionless coordinate; $\beta (\chi )$ is the implicit function which satisfies the following equation:

$$\begin{aligned} \beta \tan (\beta ) = \chi ^{-1}. \end{aligned}$$

**Figure 3:**
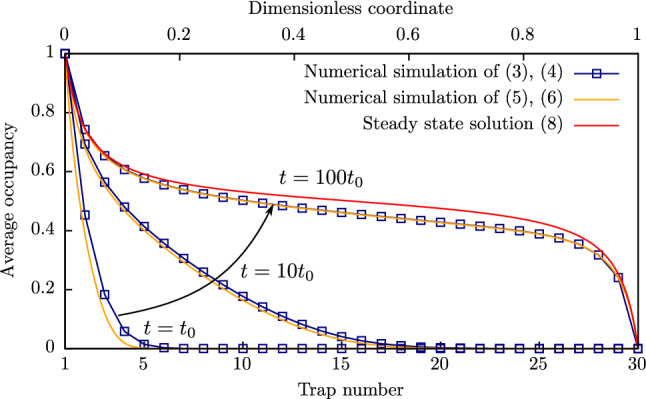
Average occupancy of traps in the 30-trap chain at different moments obtained by the numerical simulation of Eqs. (3) and (4), obtained by the numerical simulation of Eqs. (5) and (6), and the average occupancy according to the steady-state solution (8).

The solution (8) now can be used to obtain stationary current values (which are, in most cases, the final objective of the study):

$$\begin{aligned} j_{{{\text {stat}}}} = q N \dfrac{a}{4} \left[ {\text {Tun}}^+ - {\text {Tun}}^-\right] \left[ 1 + \tan ^{-2}\beta (\Omega )\right] . \end{aligned}$$

Analytical stationary solutions can be found in a general case for boundary conditions (6):

9 $$\begin{aligned} {\bar{n}}_{{\text {stat}}} = \dfrac{1}{2} {\left\{ \begin{array}{ll} 1 - B \sqrt{|k|} \tan \left( {\tilde{x}}\sqrt{|k|} - \tan ^{-1}(f_0(k))\right) , &{}\quad \text {for } k \geqslant 0,\\ 1 + B \sqrt{|k|} \coth \left( {\tilde{x}}\sqrt{|k|} + \coth ^{-1}(f_0(k))\right) , &{}\quad \text {for } k<0,\; |f_0(k)| \geqslant 1,\\ 1 + B \sqrt{|k|} \tanh \left( {\tilde{x}}\sqrt{|k|} + \tanh ^{-1}(f_0(k))\right) , &{}\quad \text {for } k<0,\; |f_0(k)| < 1,\\ \end{array}\right. } \end{aligned}$$

where *k* is the transcendental equation root:

$$\begin{aligned} f_1(k') = {\left\{ \begin{array}{ll} -\tan \left( \sqrt{|k'|} - \tan ^{-1}(f_0(k'))\right) , &{}\quad \text {for } k' \geqslant 0,\\ \coth \left( \sqrt{|k'|} + \coth ^{-1}(f_0(k'))\right) , &{}\quad \text {for } k'< 0,\; |f_0(k')| \geqslant 1,\\ \tanh \left( \sqrt{|k'|} + \tanh ^{-1}(f_0(k'))\right) , &{}\quad \text {for } k'< 0,\; |f_0(k')| < 1.\\ \end{array}\right. } \end{aligned}$$

The constant *B* and the functions $f_0(k)$, $f_1(k)$ are following:

$$\begin{aligned} B&= \dfrac{a}{d} \dfrac{{\text {Tun}}^+ + {\text {Tun}}^-}{{\text {Tun}}^+ - {\text {Tun}}^-},\quad f_0(k) = \dfrac{D_0 - (1 + k B^2)C_0}{\sqrt{|k|}},\quad f_1(k) = \dfrac{D_1 + (1 + k B^2)C_1}{\sqrt{|k|}},\\ C_\chi&= \dfrac{d}{2 a} \dfrac{({\text {Tun}}^+ - {\text {Tun}}^-)^2}{({\text {Inj}}+ {\text {Ion}})({\text {Tun}}^+ + {\text {Tun}}^-)} \Big |_{{\tilde{x}} = \chi },\quad D_\chi = \dfrac{d}{a} \dfrac{({\text {Inj}}- {\text {Ion}})({\text {Tun}}^+ - {\text {Tun}}^-)}{({\text {Inj}}+ {\text {Ion}})({\text {Tun}}^+ + {\text {Tun}}^-)} \Big |_{{\tilde{x}} = \chi }, \end{aligned}$$

The solution (9) now can be used to obtain stationary current values:

10 $$\begin{aligned} j_{{{\text {stat}}}} = q N \dfrac{a}{4} \left[ {\text {Tun}}^+ - {\text {Tun}}^-\right] \left[ 1 + B^2 k\right] . \end{aligned}$$

Despite its analytical complexity, the obtained solution (9) and (10) can be indispensable for verification of various numerical codes.

Since, the solution (8) is more simple, it can be analysed as follows. In Fig. 3 is a comparison of obtained numerical solutions using different approaches at different moments for the 30-trap chain. A scheme of linear differences is presented by blue symbols, the solution of differential equation (5) is shown by orange lines and the steady-state solution (8) is shown by a red line. The case of bulk-limited current is considered. It should be noted, that the non-stationary solution becomes reasonably close to the steady-state one at $t \gtrsim 100 t_0$.

**Figure 4:**
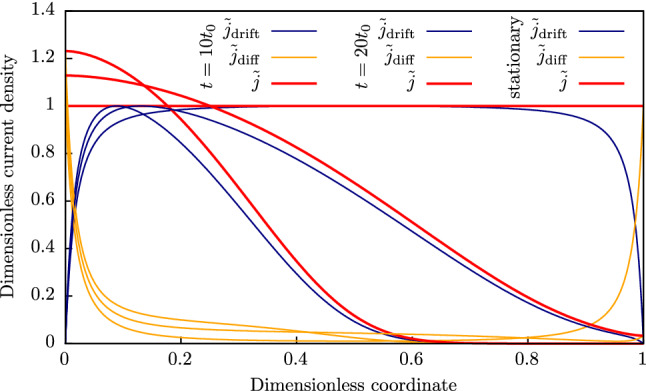
Dimensionless current density (${{\tilde{j}} = j/j_{\text {stat}}}$) distribution over dielectric bulk at different moments and stationary dimensionless current density.

**Figure 5:**
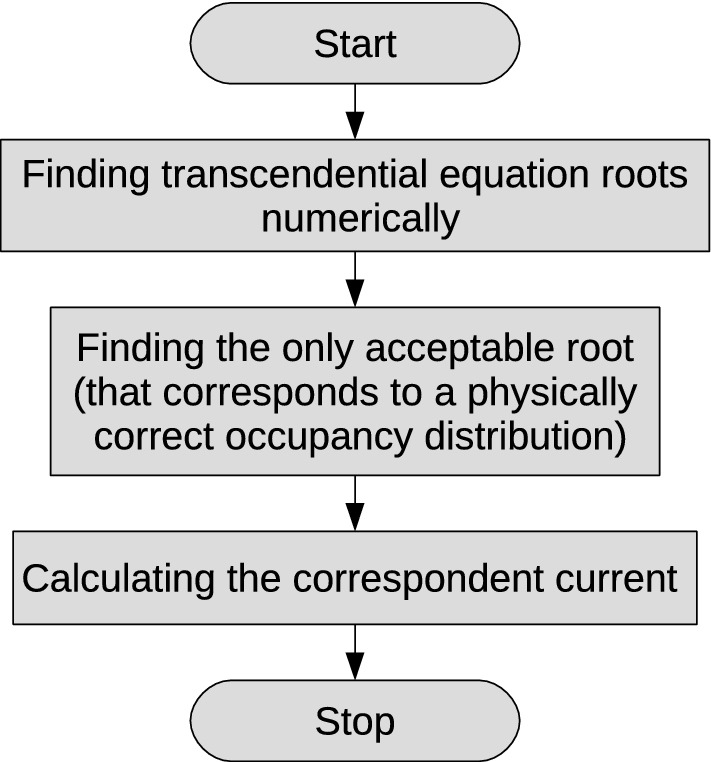
The flowchart of current distributions over a dielectric bulk simulations in stationary case.

The current distributions over a dielectric bulk at different moments are shown in Fig. 4. Figure 5 shows the flowchart for stationary case simulations. The current distributions for non-stationary cases were calculated by solving Eqs. (5)–(7) within standard integration techniques. All simulation parameters are shown in Table 2. One can see that the drift current is mostly dominant in the bulk, while the diffusion one reaches significant values near contacts. This means that the diffusion contribution can not be neglected, especially, in case of strongly out-of-balance (transient) states that take place in real processes in modern ultra-fast microelectronic devices^32^. As expected, the total current does not depend on the coordinate in the stationary case.

It should be noted, that it is not possible to measure $j_{{{\text {drift}}}}$ and $j_{{{\text {diff}}}}$ separately, only $j_{{{\text {total}}}}$ can be measured in real experiments. However, it is possible to calculate $j_{{{\text {drift}}}}$ and $j_{{{\text {diff}}}}$. The algorithm is following:

1. measure *J*-*F*-*T* characteristics;
2. fit simulated curves with experimental ones using any microscopic models of injection from contacts and trap capture/emission processes, obtain the trap parameters (trap ionisation energy, trap density, attempt-to-escape rate etc.);
3. calculate ${\bar{n}}$ distributions over the dielectric bulk at different voltages using Eq. (9) and obtained trap parameters;
4. calculate $j_{\text {drift}}$ and $j_{{{\text {diff}}}}$ distributions over the dielectric bulk at different voltages using Eq. (10) and obtained trap parameters.

**Table 2:** Parameters used in the continuum simulation.

${\text {Tun}}^-(x)$	${\text {Tun}}^+(x)$	$t_0$	${\bar{n}}(0)$	${\bar{n}}(d)$
0	*P* (any, e.g. 1)	1/*P*	1	0

Equation (5) has an obvious generalization for the case:

11 $$\begin{aligned} \frac{\partial {\bar{n}}}{\partial t} = \sum _{i = 1}^{3} \left[ - a \frac{\partial }{\partial x_{i}} \left\{ \left[ {\text {Tun}}(F_{i}) - {\text {Tun}}(-F_{i})\right] {\bar{n}}\left( 1 - {\bar{n}}\right) \right\} +\dfrac{a^2}{2} \frac{\partial }{\partial x_{i}} \left\{ \left[ {\text {Tun}}(F_{i}) + {\text {Tun}}(-F_{i})\right] \frac{\partial {\bar{n}}}{\partial x_{i}}\right\} \right] . \end{aligned}$$

Here *i* is the coordinate index. Equation (11) can be written in a vector form as follows:

$$\begin{aligned} \frac{\partial {\bar{n}}}{\partial t} = - a {\varvec{\nabla }}\cdot \left\{ \left[ {{\varvec{P}}}_{\text {tun}}^+ - {{\varvec{P}}}_{\text {tun}}^-\right] {\bar{n}}\left( 1 - {\bar{n}}\right) \right\} +\dfrac{a^2}{2} {\varvec{\nabla }}\cdot \left\{ \left[ {{\varvec{P}}}_{\text {tun}}^+ + {{\varvec{P}}}_{\text {tun}}^-\right] \circ {\varvec{\nabla }}{\bar{n}}\right\} , \end{aligned}$$

where ${{\varvec{P}}}_{\text {tun}}^\pm = ({\text {Tun}}^\pm (F_{x}), {\text {Tun}}^\pm (F_{y}), {\text {Tun}}^\pm (F_{z}))$ is a vector of tunnelling probability, the symbol $\circ$ defines the Hadamard product (also known as the Schur product or the entrywise product):

$$\begin{aligned} (A\circ B)_{ij}=(A)_{ij}(B)_{ij}, \end{aligned}$$

where *A* and *B* are two matrices (vectors) of the same dimension.

### Model of phonon-coupled traps in dielectrics

#### Phonon-assisted tunnelling of electrons (holes) between neighbouring traps

**Figure 6:**
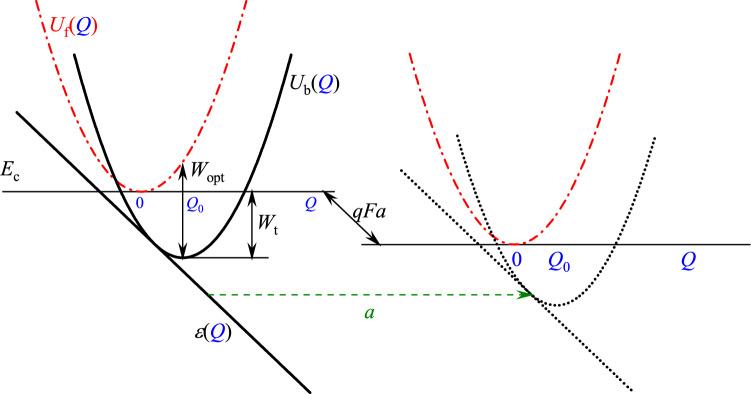
Configuration diagram for two phonon-coupled traps. $U_{\text {f}}(Q)$ is the potential energy of an empty oscillator (without trapped electron); $U_{\text {b}}(Q)$ is the potential energy of an occupied oscillator (with trapped electron); $\varepsilon (Q)$ is the position of the energy level of the trapped electron dependent on coordinate *Q*; and $E_{\text {c}}$ is the conduction-band edge. The solid and dotted lines refer to the initially occupied and empty state, respectively. The most probable tunnelling transition for the electron when both oscillators take position $Q_0/2$ is shown by the horizontal dashed arrow.

Recently, it has been shown, that the PATENT model adequately describes the current in high-$\kappa$ dielectrics^30,33–35^. An energy diagram of the electron tunnelling from a phonon-coupled trap to the other one at a distance of *a* in an external electric field is shown in Fig. 6. The energy dependency from configuration coordinate *Q* of a system trapped-electron-plus-phonons is shown by $U_{\text {b}}(Q)$ curve. The $U_{\text {f}}(Q)$ curve corresponds to a “free” electron in the conduction band. Solid lines show the initial state (before the tunnelling act), dashed lines represent the final state (after the tunnelling act). Due to the effect of the external electric field, electrons localized on the neighbouring traps have different energies represented by slanted lines $\varepsilon (Q)$ in Fig. 6, and the tunnel event must be accompanied by inelastic processes, like phonon emission and/or absorption to compensate the energy difference. All the above is taken into account in the PATENT model.

According to the PATENT model, the tunnel junction rate between neighbouring traps is the following:

12 $$\begin{aligned} {\text {Tun}}&= \int \limits _{\varepsilon >0} \dfrac{\hslash \varepsilon }{m^*a^2 kTQ_0} \exp \left( -\dfrac{(Q-Q_0)^2 - (Q-qFa/Q_0)^2}{2kT}\right) \exp \left( -\dfrac{4}{3}\dfrac{\sqrt{2m^*}(\varepsilon ^{3/2} -(\varepsilon -qFa)^{3/2})}{qF\hslash }\right) {\text{d}}Q, \\&\quad \varepsilon = Q_0(Q-Q_0)+W_{\text {opt}},\qquad Q_0=\sqrt{2(W_{\text {opt}}-W_{\text {t}})}, \end{aligned}$$

where $\hslash$ is the Dirac constant, $m^*$ is the effective mass of trapped electron (hole), *k* is the Boltzmann constant, *T* is temperature, *q* is the elementary charge (absolute value), $W_{\text {t}}$ and $W_{\text {opt}}$ are thermal and optical trap energy, respectively, and $Q_0$ is the configuration coordinate that characterize the electron-phonon interaction.

In case of low electric fields $qFa\ll W_{\text {t}}$, Eq. (12) can be simplified to

13 $$\begin{aligned} {\text {Tun}}= \dfrac{2\sqrt{\pi }\hslash W_{\text {t}}}{m^*a^2 Q_0 \sqrt{kT}} \exp \left( -\dfrac{W_{\text {opt}}- W_{\text {t}}}{2kT}\right) \exp \left( -\dfrac{2a\sqrt{2m^*W_{\text {t}}}}{\hslash }\right) \sinh \left( \frac{qFa}{2kT}\right) , \end{aligned}$$

where, the pre-exponent ratio is the attempt-to-escape rate, the first exponent is the thermal ionization effect (with the activation energy of $(W_{\text {opt}}-W_{\text {t}})/2$), the second exponent is the tunnelling factor and the hyperbolic sine represent the activation energy decreasing due to the external electric field with respect to transitions in both co-field and contrafield directions.

The analysis shows that, in non-low electric fields ($qFa\geqslant 1/4W_{\text {t}}$), Eq. (13) gives a large deviation from experiment data, and Eq. (12) should be applied instead.

#### Ionization of phonon-coupled traps to contacts

Ionization of phonon-coupled traps to contacts is described according to Refs. ^29,36^ by the following equation:

14 $$\begin{aligned} {\text {Ion}}_F= & {} \int \limits _{\begin{array}{c} \varepsilon>0, \\ \varepsilon -qFa^{*}>0 \end{array}} \frac{V_{\text {out}}}{2a^{*}\sqrt{2\pi kT}} \exp \left( -\frac{(Q-Q_0)^2}{2kT}\right) \exp \left( -\frac{4}{3}\frac{\sqrt{2m^*}(\varepsilon ^{3/2}-(\varepsilon -qFa^{*})^{3/2})}{qF\hslash }\right) \nonumber \\&\times \left( 1-f_{\text {F-D}}\left( \Phi -\varepsilon +qFa^{*}\right) \right) {\text{d}}{Q}. \end{aligned}$$

Here $\Phi$ is difference between the energy of the dielectric conduction band bottom and the Fermi level in the contact, $a^{*}$ is the trap-to-contact distance, $V_{\text {out}}$ the free electron velocity in a contact, $f_{\text {F-D}}({\tilde{E}})$ is the Fermi–Dirac distribution function, ${\tilde{E}}$ is the electron energy relative to the Fermi level.

Additionally, thermal ionization, which takes place in case of zero electric field $F=0$, should be taken into account:

15 $$\begin{aligned} {\text {Ion}}_T = \int \limits _{\varepsilon >0} \frac{V_{\text {out}}}{2a^{*}\sqrt{2\pi kT}} \exp \left( -\frac{(Q-Q_0)^2}{2kT}\right) \exp \left( -\frac{2a^{*}\sqrt{2m^*\varepsilon }}{\hslash }\right) \left( 1-f_{\text {F-D}}\left( \Phi -\varepsilon \right) \right) {\text{d}}{Q}. \end{aligned}$$

The total ionization rate is composed of terms (14) and (15):

16 $$\begin{aligned} {\text {Ion}}= {\left\{ \begin{array}{ll} {\text {Ion}}_F, &{} \quad \text {for } F \ne 0,\\ {\text {Ion}}_T, &{} \quad \text {for } F = 0. \end{array}\right. } \end{aligned}$$

#### Injection from contacts to phonon-coupled traps

The injection rate ${\text {Inj}}$ from the contact to the trap is similar to Eqs. (14)–(16), taking into account that the electron is injected from the occupied state:

17 $$\begin{aligned} {\text {Inj}}_F= & {} \int \limits _{\begin{array}{c} \varepsilon>0, \\ \varepsilon -qFa^{*}>0 \end{array}} \frac{V_{\text {out}}}{2a^{*}\sqrt{2\pi kT}} \exp \left( -\frac{(Q-Q_0)^2}{2kT}\right) \exp \left( -\frac{4}{3}\frac{\sqrt{2m^*}(\varepsilon ^{3/2}-(\varepsilon -qFa^{*})^{3/2})}{qF\hslash }\right) f_{\text {F-D}}\left( \Phi -\varepsilon +qFa^{*}\right) {\text{d}}{Q},\end{aligned}$$

18 $$\begin{aligned} {\text {Inj}}_T= & {} \int \limits _{\varepsilon >0} \frac{V_{\text {out}}}{2a^{*}\sqrt{2\pi kT}} \exp \left( -\frac{(Q-Q_0)^2}{2kT}\right) \exp \left( -\frac{2a^{*}\sqrt{2m^*\varepsilon }}{\hslash }\right) f_{\text {F-D}}\left( \Phi -\varepsilon \right) {\text{d}}{Q},\end{aligned}$$

19 $$\begin{aligned} {\text {Inj}}= & {} {\left\{ \begin{array}{ll} {\text {Inj}}_F, &{} \quad \text {for } F \ne 0,\\ {\text {Inj}}_T, &{} \quad \text {for } F = 0. \end{array}\right. } \end{aligned}$$

### Comparison with experiments

The theoretical results were compared to the data obtained in the experiment on measuring the conductivity of amorphous $\hbox {HfO}_2$ films deposited in the ALD process. In the experiment, the electric current *versus* the electric field applied to the 20-nm-thick dielectric at different temperatures were measured.

**Figure 7:**
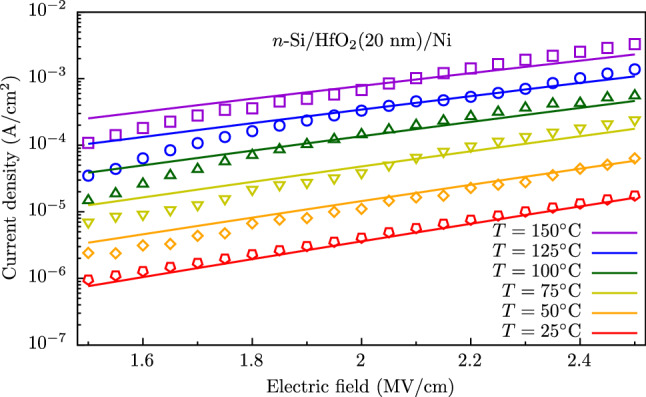
Experimental current-field characteristics (symbols) of the *n*-Si/$\hbox {HfO}_2$/Ni structure^30^ and simulation (lines) in terms of the PATENT model at different temperatures.

The measured current-field characteristics *J*-*F*-*T* are shown by symbols in Fig. 7. The calculated *J*-*F*-*T* characteristics using equations (9), (10), (12), (14)–(19) are represented by lines in Fig. 7. One can see that the calculations in terms of a developed model are in a good quantitative agreement with the experimental data. In the calculations, the electron barriers on contacts as adopted parameters were used as breaks in the conduction band bottom in the contacts and dielectric $\Phi _{{{{\text{Si}}}}/{{\text{HfO}}}_2}$ and $\Phi _{{{\text{HfO}}}_2/{{\text{Ni}}}}$ that are in agreement with the literature data^35,37^, thermal and optic trap energies are corresponds to an oxygen vacancy in $\hbox {HfO}_2$^30,35^, the mean distance between traps $a=a^*=1.8\,\hbox {nm}$ corresponds to the total trap density $N=1.7\times 10^{20}\,\hbox {cm}^{-3}$ that is close to value $2.5\times 10^{20}\,\hbox {cm}^{-3}$ obtained in the original article^30^, while the effective electron mass $m^*=0.59m_0$ (here $m_0$ is the free electron mass) is closer to the values calculated within *ab initio* simulations^38^ than the original value $m^*=0.8m_0$ that was obtained when ignoring the diffusion contribution^30^. All simulation parameter values are shown in Table 3.

**Table 3:** Physical parameters used in the simulation.

Material	$\Phi _{\text {Material}/{\text{HfO}}_2}$ (eV)	*d* (nm)	*N* ($\hbox {cm}^{-3}$)	$W_{\text {t}}$ (eV)	$W_{\text {opt}}$ (eV)	$m^*/m_0$
$\hbox {HfO}_2$	–	20	$(1.7\pm 0.1)\times 10^{20}$	1.25	2.5	$0.59\pm 0.3$
Si	1.9	–	–	–	–	–
Ni	2.5	–	–	–	–	–

## Discussion

We have developed a continuum model of the charge transport in dielectrics, which takes into account the drift and diffusion contributions to the trapped charge carriers current. The developed continuum model is universal, i.e. it can be supplemented with a different injection from contacts and trap capture/emission microscopic processes, including Hill-Adachi, PATENT, TAT and other trap ionisation models. It is shown that neither of contributions can be neglected in case of ultrathin dielectric films. The analytical stationary solutions, that are close to experimentally achievable conditions, are found. A comparison with other theoretical models and experiments shows a good agreement. The model can be extended to two- and three-dimensional systems. The developed model, formulated in partial differential equations, can be implemented in the finite element method code that is compatible with other partial differential equations, e.g. Poison and thermal equations. The joint solution of these equations can significantly advance the search of optimal parameters for electronic devices and conditions for their fabrication, including promising ones: RRAM, FRAM and many others. The found analytical solutions will be useful for the verification of various numerical codes for simulations of physics of electronic devices, including stress induced leakage currents in FETs and flash memories, currents during the resistive switching of RRAM and memristors and leakage currents in FRAM devices.

## Methods

### Preparation of samples for transport measurements

Transport measurements were performed for metal-insulator-semiconductor (MIS). For the MIS Si/$\hbox {HfO}_2$/Ni structures, the 20-nm-thick amorphous hafnia was deposited on a *n*-type Si (100) wafer by using the atomic layer deposition (ALD) system as described according to Ref. ^30^. Tetrakis dimethyl amino hafnium (TDMAHf) and water vapour were used as precursors at a chamber temperature of $250^{\circ }\hbox {C}$ for $\hbox {HfO}_2$ film deposition. The samples for transport measurements were equipped with round 50-nm-thick Ni gates with a radius of $70\,\mu \hbox {m}$.

### Transport measurements

Transport measurements were performed using a Hewlett Packard 4155B semiconductor parameter analyzer and an Agilent E4980A precision LCR meter.
